# Supplementary material for: Inverted U-Shaped Relationship between Obesity Parameters and Bone Mineral Density in Korean Adolescents
Source: J Clin Med. 2023 Sep 9;12(18):5869. doi: 10.3390/jcm12185869 (PMC10532009; doi:10.3390/jcm12185869)
Supplement: Supplementary file 1 [file jcm-12-05869-s001.zip › jcm-2563678-supplementary.pdf]

Online-only Supplementary Materials

Inverted U-shaped Relationship between Obesity Parameters and Bone Mineral Density in Korean Adolescents

Jongseok Lee[1,*], Insang Yoon[1], Hwajung Cha[1], Ho-Jung Kim[1], Ohk-Hyun Ryu[2,*]

[1] School of Artificial Intelligence Convergence, Hallym University, Chuncheon 24253, Korea; ljs1844@hallym.ac.kr; pny235711@gmail.com; nataliac@kakao.com; hojungkim@hallym.ac.kr; ohryu30@gmail.com

[2] Division of Endocrinology and Metabolism, Department of Internal Medicine, Chuncheon Sacred Heart Hospital, Chuncheon 24253, Korea; ohryu30@gmail.com

*** Corresponding Author:**

Jongseok Lee, Ph.D.

School of Artificial Intelligence Convergence, Hallym University, Chuncheon 24253, Korea

ljs1844@hallym.ac.kr; Tel.: +82-33-248-3585

Ohk-Hyun Ryu, M.D. Ph.D.

Division of Endocrinology and Metabolism, Department of Internal Medicine, Chuncheon

Sacred Heart Hospital, Chuncheon 24253, Korea

ohryu30@gmail.com; Tel.: +82-33-240-5833

Content list:

Supplementary Table S1. Gender- and age-specific 5th, 85th, and 95th percentiles of body mass index (BMI) based on the 2007 Korea National Growth Chart

Supplementary Table S2. Sample distribution of gender-and-age-specific percentile groups of body mass index (BMI) by gender and age

Supplementary Table S3. Sample distribution of gender-specific percentile groups of waist circumference (WC), waist-to-height ratio (WHtR), and total-body fat mass (FM) by gender

Supplementary Table S4. Sample distribution according to age groups, overall and by gender

Supplementary Table S5. Mean comparison between male and female by age group

Supplementary Table S6. Mean comparison of age among four percentile groups of each obesity parameter by gender in 2,060 Korean adolescents aged 10 to 19 years from the KNHANES 2008-2011

Supplementary Table S7. Mean comparison of total-body-less-head (TBLH) bone mineral density (BMD) among four percentile groups of each obesity parameter by gender in 2,060 Korean adolescents aged 10 to 19 years from the KNHANES 2008-2011

Supplementary Figure S1. Non-linear relationship between obesity and the estimated values of total-body-less-head (TBLH) bone mineral density (BMD), while adjusting for age, menarcheal status (for female only), and total-body lean mass, by gender in 2,060 Korean adolescents aged 10 to 19 years from the 2008-2011 Korea National Health and Nutrition Examination Survey (KNHANES).

Supplementary Table S1. Gender- and age-specific 5th, 85th, and 95th percentiles of body mass index (BMI) based on the 2007 Korea National Growth Chart

		Age (years)								
Gender	BMI Percentiles*	10	11	12	13	14	15	16	17	18
Male	5th percentile (kg/m^2)	14.57	14.93	15.35	15.82	16.32	16.83	17.33	17.80	18.20
	85th percentile (kg/m^2)	21.71	22.57	23.32	23.93	24.40	24.74	24.95	25.08	25.18
	95th percentile (kg/m^2)	24.48	25.50	26.35	27.02	27.48	27.77	27.89	27.89	27.85
Female	5th percentile (kg/m^2)	14.33	14.73	15.20	15.71	16.25	16.78	17.27	17.68	17.96
	85th percentile (kg/m^2)	20.71	21.51	22.22	22.83	23.31	23.67	23.89	23.99	23.98
	95th percentile (kg/m^2)	23.08	23.99	24.77	25.38	25.83	26.11	26.24	26.24	26.15

* The BMI percentile groups were stratified based on the 2007 Korea National Growth Charts [38]

Supplementary Table S2. Sample distribution of gender-and-age-specific percentile groups of body mass index (BMI) by gender and age

		Age (years)										
		10	11	12	13	14	15	16	17	18	19	Total
Gender	BMI Percentiles*	*n*	*n*	*n*	*n*	*n*	*n*	*n*	*n*	*n*	*n*	*n*
Male	< 5th	6	3	8	8	8	7	12	6	13	5	76
	5th – 85th	82	98	99	79	105	86	59	83	61	69	821
	86th – 94th	24	14	18	16	14	10	13	12	8	8	137
	≥ 95th	6	12	8	6	16	3	9	6	4	10	80
	Total	118	127	133	109	143	106	93	107	86	92	1,114
Female	< 5th	6	5	5	2	7	5	7	7	10	12	66
	5th – 85th	86	81	63	85	70	59	65	74	35	75	693
	86th – 94th	13	13	14	20	17	11	7	7	5	11	118
	≥ 95th	2	8	7	8	9	3	7	7	6	12	69
	Total	107	107	89	115	103	78	86	95	56	110	946

* The BMI percentile groups were stratified based on the 2007 Korea National Growth Charts [38]

Supplementary Table S3. Sample distribution of gender-specific percentile groups of waist circumference (WC), waist-to-height ratio (WHtR), and total-body fat mass (FM) by gender

		WC (cm)		WHtR (cm/cm)		Total-body FM (kg)	
Gender	Percentiles*	Range	*n*	Range	*n*	Range	*n*
Male (*n* = 1,114)	< 5th	45.9 – 56.0	55	0.313 – 0.357	56	3.732 – 5.531	55
	5th – 85th	56.1 – 82.5	897	0.358 – 0.494	891	5.539 – 20.687	892
	86th – 94th	82.6 – 90.2	109	0.495 – 0.542	112	20.715 – 26.656	112
	≥ 95th	90.3 – 116.1	53	0.543 – 0.694	55	26.696 – 46.751	55
Female (*n* = 946)	< 5th	46.0 – 55.2	49	0.326 – 0.362	47	4.660 – 7.884	47
	5th – 85th	55.3 – 76.0	756	0.363 – 0.477	757	7.886 – 22.266	757
	86th – 94th	76.1 – 83.0	95	0.478 – 0.517	95	22.316 – 27.193	95
	≥ 95th	83.1 – 115.0	46	0.518 – 0.689	47	27.231 – 46.827	47

WC: waist circumference; WHtR: waist-to-height ratio; FM: fat mass. * The percentile groups of WC, WHtR, and total-body FM were determined using the observed sample distributions in the study, separately for males and females.

Supplementary Table S4. Sample distribution according to age groups, overall and by gender

		Age (years)										
		10	11	12	13	14	15	16	17	18	19	Total
Gender												
Male	*n*	118	127	133	109	143	106	93	107	86	92	1,114
	(%)	(10.6)	(11.4)	(11.9)	(9.8)	(12.8)	(9.5)	(8.3)	(9.6)	(7.7)	(8.3)	(100)
Female	*n*	107	107	89	115	103	78	86	95	56	110	946
	(%)	(11.3)	(11.3)	(9.4)	(12.2)	(10.9)	(8.2)	(9.1)	(10.0)	(5.9)	(11.6)	(100)
Total	*n*	225	234	222	224	246	184	179	202	142	202	2,060
	(%)	(10.9)	(11.4)	(10.8)	(10.9)	(11.9)	(8.9)	(8.7)	(9.8)	(6.9)	(9.8)	(100)

Supplementary Table S5. Mean comparison between male and female by age group

Variable	Age (years)	Male (*n* = 1,114) *n*	Mean	(95% CI)	Female (*n* = 946) *n*	Mean	(95% CI)	*p*-value*
TBLH BMD (g/cm^2)	10	118	0.708	(0.696, 0.719)	107	0.703	(0.688, 0.718)	0.604
	11	127	0.759	(0.745, 0.772)	107	0.757	(0.741, 0.773)	0.901
	12	133	0.805	(0.791, 0.818)	89	0.814	(0.797, 0.832)	0.398
	13	109	0.851	(0.833, 0.868)	115	0.842	(0.828, 0.857)	0.483
	14	143	0.930	(0.912, 0.947)	103	0.849	(0.835, 0.864)	< 0.001
	15	106	0.941	(0.925, 0.958)	78	0.870	(0.853, 0.888)	< 0.001
	16	93	0.972	(0.949, 0.994)	86	0.880	(0.863, 0.897)	< 0.001
	17	107	0.984	(0.966, 1.002)	95	0.880	(0.863, 0.896)	< 0.001
	18	86	1.006	(0.983, 1.030)	56	0.878	(0.858, 0.897)	< 0.001
	19	92	1.022	(1.002, 1.041)	110	0.905	(0.891, 0.920)	< 0.001
TB Lean mass (kg)	10	118	25.74	(25.03, 26.45)	107	24.13	(23.35, 24.92)	0.003
	11	127	29.70	(28.72, 30.69)	107	28.34	(27.42, 29.25)	0.048
	12	133	33.68	(32.56, 34.80)	89	31.60	(30.60, 32.61)	0.007
	13	109	39.04	(37.72, 40.36)	115	33.15	(32.30, 34.01)	< 0.001
	14	143	44.74	(43.66, 45.82)	103	33.37	(32.48, 34.25)	< 0.001
	15	106	46.46	(45.20, 47.71)	78	33.87	(32.95, 34.80)	< 0.001
	16	93	48.36	(46.97, 49.75)	86	33.99	(33.13, 34.84)	< 0.001
	17	107	49.91	(48.62, 51.20)	95	34.42	(33.47, 35.37)	< 0.001
	18	86	48.85	(47.60, 50.11)	56	34.46	(33.16, 35.77)	< 0.001
	19	92	51.27	(49.84, 52.71)	110	35.32	(34.51, 36.13)	< 0.001
TB fat mass (kg)	10	118	11.36	(10.36, 12.35)	107	11.32	(10.60, 12.04)	0.954
	11	127	13.05	(11.87, 14.23)	107	13.35	(12.33, 14.38)	0.707
	12	133	13.65	(12.43, 14.88)	89	14.58	(13.40, 15.75)	0.282
	13	109	13.27	(12.01, 14.52)	115	15.98	(14.92, 17.03)	0.001
	14	143	14.12	(12.81, 15.43)	103	17.84	(16.73, 18.96)	< 0.001
	15	106	12.93	(11.62, 14.24)	78	17.29	(16.07, 18.51)	< 0.001
	16	93	14.22	(12.67, 15.77)	86	18.33	(17.04, 19.62)	< 0.001
	17	107	14.30	(13.02, 15.59)	95	18.77	(17.53, 20.01)	< 0.001
	18	86	13.54	(12.27, 14.81)	56	19.03	(17.18, 20.88)	< 0.001
	19	92	14.00	(12.57, 15.42)	110	18.27	(17.15, 19.39)	< 0.001

CI: confidence interval; TBLH: total-body-less-head; BMD: bone mineral density; TB: total-body. *Statistical significances were assessed using *t*-test for two independent samples.

Supplementary Table S6. Mean comparison of age among four percentile groups of each obesity parameter by gender in 2,060 Korean adolescents aged 10 to 19 years from the KNHANES 2008-2011

		Mean (SD) of Age (years) in four percentile groups based on each obesity parameter*								
		Underweight ($< 5^{th}$)		Normal-weight ($5^{th} – 85^{th}$)		Overweight ($86^{th} – 94^{th}$)		Obesity ($\geq 95^{th}$)		
Gender	Obesity parameter	Mean	(SD) $^{T \dagger}$	Mean	(SD) $^{T \dagger}$	Mean	(SD) $^{T \dagger}$	Mean	(SD) $^{T \dagger}$	*p*-value‡
Male	Body mass index (kg/m^2)	14.91	(2.69) ^{b}	14.17	(2.80) $^{a, b}$	13.66	(2.82) ^{a}	14.29	(2.85) $^{a, b}$	0.085
	Waist circumference (cm)	11.40	(1.76) ^{a}	14.13	(2.78) ^{b}	15.28	(2.63) ^{c}	15.47	(2.35) ^{c}	< 0.001
	Waist-to-height ratio (cm/cm)	14.54	(2.29) ^{b}	14.27	(2.81) $^{a, b}$	13.52	(2.87) $^{a, b}$	13.42	(2.83) ^{a}	0.001
	Total-body fat mass (kg)	13.11	(2.63) ^{a}	14.17	(2.84) $^{a, b}$	14.50	(2.76) ^{b}	14.47	(2.28) ^{b}	0.016
Female	Body mass index (kg/m^2)	15.35	(3.04) ^{c}	14.18	(2.91) $^{a, b}$	13.85	(2.70) ^{a}	14.99	(2.88) $^{b, c}$	0.678
	Waist circumference (cm)	11.39	(1.78) ^{a}	14.28	(2.88) ^{b}	15.22	(2.75) $^{b, c}$	15.54	(2.66) ^{c}	< 0.001
	Waist-to-height ratio (cm/cm)	13.33	(2.44) ^{a}	14.26	(2.91) $^{a, b}$	14.75	(2.91) ^{b}	14.89	(2.98) ^{b}	0.002
	Total-body fat mass (kg)	11.36	(1.65) ^{a}	14.23	(2.90) ^{b}	15.33	(2.64) $^{b, c}$	15.98	(2.62) ^{c}	< 0.001

KNHANES: Korea National Health and Nutrition Examination Survey; SD: standard deviation; BMI: body mass index; WC: waist circumference; WHtR: waist-to-height ratio; FM: fat mass.

* The gender- and age-specific percentile groups of BMI were stratified on the basis of the 2007 Korea National Growth charts [38] (Supplementary Table 1 and 2), and the gender-specific percentile groups of WC, WHtR, and total-body FM were determined using the observed sample distributions in the study, separately for males and females (Supplementary Table 3)

† Mean (SD) was calculated by one-way analysis of variances (ANOVA). The same letters indicate that the mean difference between groups is not significant ($\alpha = 0.05$) on pairwise post-hoc comparisons by the bonferroni test (equal variance assumed) or the Games Howell test (equal variance not assumed).

‡ Statistical significances were evaluated to test for whether there is a significant linear trend for the means of age to increase across the percentile groups of each obesity parameter.

Supplementary Table S7. Mean comparison of total-body-less-head (TBLH) Bone mineral density (BMD) among four percentile groups of each obesity parameter by gender in 2,060 Korean adolescents aged 10 to 19 years from the KNHANES 2008-2011

		Mean (SD) of TBLH BMD (g/cm^2) in four percentile groups based on each obesity parameter*								
		Underweight (< 5th)		Normal-weight (5th – 85th)		Overweight (86th – 94th)		Obesity (≥ 95th)		
Gender	Obesity parameter	Mean	(SD) T †	Mean	(SD) T †	Mean	(SD) T †	Mean	(SD) T †	*p*-value‡
Male	Body mass index (kg/m^2)	0.803	(0.111) *a*	0.885	(0.137) *b*	0.916	(0.135) *b, c*	0.942	(0.140) *c*	< 0.001
	Waist circumference (cm)	0.687	(0.059) *a*	0.884	(0.130) *b*	0.961	(0.122) *c*	1.003	(0.117) *c*	< 0.001
	Waist-to-height ratio (cm/cm)	0.817	(0.107) *a*	0.891	(0.139) *b*	0.895	(0.140) *b*	0.889	(0.132) *b*	0.060
	Total-body fat mass (kg)	0.783	(0.120) *a*	0.885	(0.137) *b*	0.932	(0.132) *b, c*	0.944	(0.119) *c*	< 0.001
Female	Body mass index (kg/m^2)	0.783	(0.101) *a*	0.824	(0.098) *b*	0.882	(0.084) *c*	0.902	(0.092) *c*	< 0.001
	Waist circumference (cm)	0.678	(0.077) *a*	0.831	(0.092) *b*	0.896	(0.086) *c*	0.919	(0.779) *c*	< 0.001
	Waist-to-height ratio (cm/cm)	0.758	(0.100) *a*	0.830	(0.098) *b*	0.879	(0.097) *c*	0.890	(0.088) *c*	< 0.001
	Total-body fat mass (kg)	0.697	(0.100) *a*	0.830	(0.093) *b*	0.892	(0.083) *c*	0.923	(0.079) *c*	< 0.001

KNHANES: Korea National Health and Nutrition Examination Survey; SD: standard deviation; TBLH: total body less head; BMD: Bone mineral density; BMI: body mass index; WC: waist circumference; WHtR: waist-to-height ratio; FM: fat mass.

* The gender- and age-specific percentile groups of BMI were stratified on the basis of the 2007 Korea National Growth charts [38] (Supplementary Table 1 and 2), and the gender-specific percentile groups of WC, WHtR, and total-body FM were determined using the observed sample distributions in the study, separately for males and females (Supplementary Table 3)

† Mean (SD) was calculated by one-way analysis of variances (ANOVA). The same letters indicate that the mean difference between groups is not significant (α = 0.05) on pairwise post-hoc comparisons by the bonferroni test (equal variance assumed) or the Games Howell test (equal variance not assumed).

‡ Statistical significances were evaluated to test for whether there is a significant linear trend for the means TBLH BMD to increase across the percentile groups of each obesity parameter.

(A) Quadratic effect of BMI on TBLH BMD

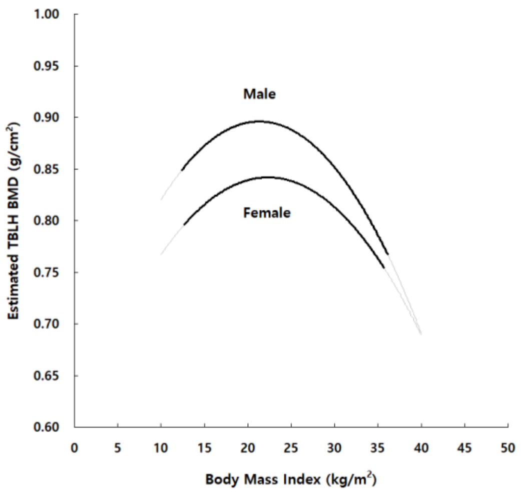

(B) Quadratic effect of WC on TBLH BMD

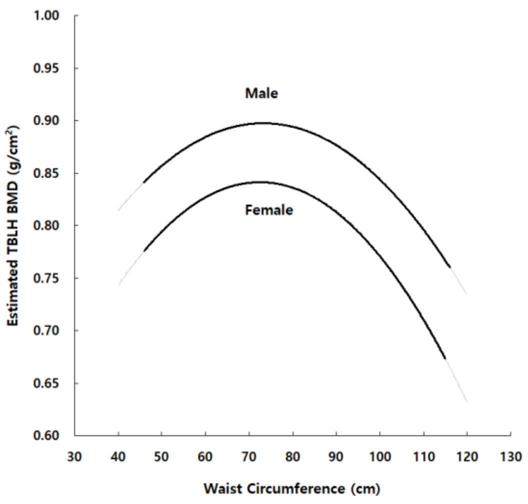

(C) Quadratic effect of WHtR on TBLH BMD

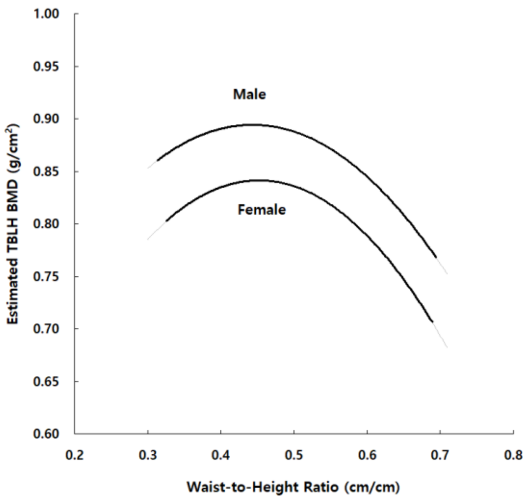

(D) Quadratic effect of FM on TBLH BMD

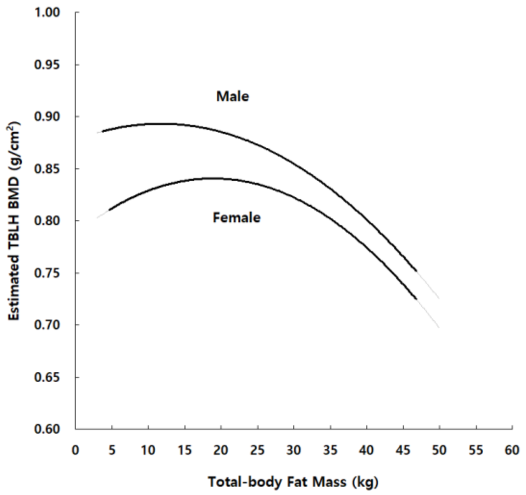

Supplementary Figure S1. Non-linear relationship between obesity and the estimated values of total-body-less-head (TBLH) bone mineral density (BMD), while adjusting for age, menarcheal status (for female only), and total-body lean mass, by gender in 2,060 Korean adolescents aged 10 to 19 years from the 2008-2011 Korea National Health and Nutrition Examination Survey (KNHANES). The graphical representation illustrates the multiple regression results, as detailed in Table 3, highlighting the quadratic relationships of TBLH BMD with four obesity parameters: (**A**) body mass index (BMI), (**B**) waist circumference (WC), (**C**) waist-to-height ratio (WHtR), and (**D**) total-body fat mass (FM). The black line indicates the estimated marginal values of TBLH BMD, considering that the covariates or confounders in the model are set at their mean values. The bold line represents the range associated with each obesity parameter.
